# Metabarcoding of the kombucha microbial community grown in different microenvironments

**DOI:** 10.1186/s13568-015-0124-5

**Published:** 2015-06-11

**Authors:** Oleg N Reva, Iryna E Zaets, Leonid P Ovcharenko, Olga E Kukharenko, Switlana P Shpylova, Olga V Podolich, Jean-Pierre de Vera, Natalia O Kozyrovska

**Affiliations:** Bioinformatics and Computational Biology Unit, Department of Biochemistry, University of Pretoria, Lynnwood road, Hillcrest, Pretoria, 0002 South Africa; Institute of Molecular Biology and Genetics of National Academy of Sciences of Ukraine, Acad. Zabolotnoho str., 150, Kiev, 03680 Ukraine; Institute of Planetary Science, DLR, Rutherfordstr. 2, 12489 Berlin, Germany

**Keywords:** Kombucha microbial community, Metabarcoding, Pyrosequencing

## Abstract

Introducing of the DNA metabarcoding analysis of probiotic microbial communities allowed getting insight into their functioning and establishing a better control on safety and efficacy of the probiotic communities. In this work the kombucha poly-microbial probiotic community was analysed to study its flexibility under different growth conditions. Environmental DNA sequencing revealed a complex and flexible composition of the kombucha microbial culture (KMC) constituting more bacterial and fungal organisms in addition to those found by cultural method. The community comprised bacterial and yeast components including cultured and uncultivable microorganisms. Culturing the KMC under different conditions revealed the core part of the community which included acetobacteria of two genera *Komagataeibacter* (former *Gluconacetobacter*) and *Gluconobacter*, and representatives of several yeast genera among which *Brettanomyces/Dekkera* and *Pichia* (including former *Issatchenkia*) were dominant. *Herbaspirillum* spp. and *Halomonas* spp., which previously had not been described in KMC, were found to be minor but permanent members of the community. The community composition was dependent on the growth conditions. The bacterial component of KMC was relatively stable, but may include additional member—lactobacilli. The yeast species composition was significantly variable. High-throughput sequencing showed complexity and variability of KMC that may affect the quality of the probiotic drink. It was hypothesized that the kombucha core community might recruit some environmental bacteria, particularly lactobacilli, which potentially may contribute to the fermentative capacity of the probiotic drink. As many KMC-associated microorganisms cannot be cultured out of the community, a robust control for community composition should be provided by using DNA metabarcoding.

**Electronic supplementary material:**

The online version of this article (doi:10.1186/s13568-015-0124-5) contains supplementary material, which is available to authorized users.

## Introduction

Culture-dependent methods have revealed an enormous microbial diversity in various fermented products. However, there is still much to be discovered about development and functioning of microbial communities. The high-throughput sequencing technologies known also as next generation sequencing (NGS) are in use to examine the phylogenetic diversity, composition, and dynamic structural changes in microbial communities of fermented foods, giving an opportunity to describe and predict relationships between species in these complex ecosystems (Kim et al. 2011; Oguntoyinbo and Narbad 2012; Park et al. 2012; Nam et al. 2012a, b; Illeghems et al. 2012; Marsh et al. 2014). Applicability of NGS for metabarcoding and metagenomic analysis of environmental DNA samples allows identifying uncultured microbial species constituting the communities. Moreover, these approaches allow coupling of structural changes in the communities with environmental factors—e.g., temperature, salinity, pH, etc.,—to perform a meta-analysis of dynamic changes of microbiota (Shade et al. 2013). DNA metabarcoding of complex bacterial and fungal communities by profiling of 16S rDNA sequences and internal transcribed regions (ITS) had opened new prospects in studying and designing of new efficient probiotics based on fermentation process.

Nowadays, when people became more concerned about obesity and prophylaxis chronic diseases, the probiotics and synbiotics have occupied an important sector within the functional food market. Most probiotic drinks are from dairy products. The tendency to veganism implied consuming of non-dairy nutraceuticals that called for design of new safe non-dairy probiotics, which became an essential health-keeping food category (Prado et al. 2008; Vasudha and Mishra 2013). Thus kombucha, in range with other fermented functional foods like kvass, fermented herb drinks, etc., may substitute dairy products for people with lactose intolerance (Gupta and Abu-Ghannam 2012). Fermented probiotic products are produced by complex microbial communities, which remain to be open environments characterized by rather unstable species composition dependent on nutritional sources and growth conditions.

Health improving effects of the kombucha probiotic beverage have been reported in a number of publications (Yapar et al. 2010; Bhattacharya et al. 2011, 2013; Aloulou et al. 2012; Kallel et al. 2012; Srihari et al. 2013). Kombucha microbial community (KMC) is an example of mutualistic metabolic cooperation of pro- and eukaryotic microorganisms (bacteria and yeasts). Several types of KMC are cultivated on different continents, which differed in the community structure and diversity (Teoh et al. 2004; Ovcharenko 2013; Marsh et al. 2014), but all of them always possessed cellulose-forming acetobacteria and yeasts. Close biochemical interplay between yeasts and bacteria was facilitated by enclosing the mixed community within cellulose-based pellicles created by the cellulose-producing bacteria on the surface of the liquid medium. The kombucha drink contains organic acids, amino acids, antibiotic substances, vitamins and also many other unidentified bioactive compounds beneficial for human health (Jayabalan et al. 2010). Kombucha was proved to exert an antimicrobial activity against pathogens (Battikh et al. 2012). Because of a relative stability of the community and the beneficial effect to human health, KCM was domesticated and widely spread around the world. It is usually cultivated in sweetened tea. Recently the kombucha and kombucha-like products with different supplements have been commercialized in many countries. It might be assumed that the KMC community is quite complex and many associated micro-organisms cannot be cultured out of the community. A robust control on the community composition might be provided by using NGS.

In this study, the microbial diversity of the kombucha variant from Ukraine (KMC-IMBG1) grown in different conditions was examined using both culture-dependent and culture-independent approaches. Study of a hybrid KMC-IMBG1 was performed to elucidate flexibility of KMC and its ability to recruit organisms from other communities in a similar way as it was reported for kimchi where additives had influenced the microbial community (Jung et al. 2011). Roche 454 pyrosequencing of amplified barcode sequences followed by a computer-based profiling of microbial species have uncovered multiple uncultivable members of KMC-IMBG1 in the pellicles and cultural liquid. KMC composition was depending on the growth conditions and showed ability to recruit accessory members such as lactobacilli.

## Materials and methods

### Microbial cultures and culturing conditions

The kombucha microbial culture was obtained from the collection of microorganisms of the Institute of Molecular Biology and Genetics of National Academy of Sciences (Kyiv, Ukraine). It was maintained in a filter sterilized black tea (Lipton, 1.2%, w/v) extract with sucrose (3.0%, w/v) (sBTS) or non-sterile BTS (nsBTS). KMC-IMBG1 also has been maintained in filter (0.22 µm, Millipore) sterilized black tea supplemented with honey (2.0%) (BTH). A matured KMC-IMBG1 was obtained after cultivation for 14 days at 28°C without shaking. A hybrid KMC was obtained by growing KMC-IMBG1 in fermented cabbage brine. More specifically, the KMC cultural liquid (10%), which previously was pre-cultured in BTH, was added to the minced cabbage supplemented with honey (2.0%). The cultivation conditions were the same as described above. Newly formed pellicles were used for inoculation of fresh BTH in a weekly basis for 5 weeks. For isolation and cultivation of acetobacteria, HS agar medium (Hestrin and Schramm 1954) was used. The isolates were incubated for 3–7 days at 30°C under stationary conditions in HS until formation of pellicles. Yeast cultures were isolated on the Glucose Yeast Peptone agar medium (HiMedia Laboratories, India). Concomitant bacteria were screened on the minimal agar medium with sucrose (Miller 1972). For medium selectivity, the antibiotics cycloheximide (100 µg/ml, Sigma-Aldrich) against yeast and ceftriaxone (50 µg/ml, Roshe) against bacteria were added to corresponding media.

### Confocal scanning laser microscopy

After cultivation for 14 days in sBTS, the bacterial cellulose-based pellicle samples were fixed in formaldehyde vapor during 1 h and stained with calcofluor (excitation 405 nm, filter BP 420–480) and thiazine red dyes (excitation 514 nm, filter BP 530–600 nm). A microscopic examination of sample fluorescence was performed, using CSLM AXIOSKOP-2 ZEISS equipped with the LSM 510 PASCAL (CarlZeiss, FRG) software.

### DNA extraction

Total DNA samples from the kombucha liquid culture and pellicle were isolated for further barcode amplification and pyrosequencing. Microbial DNA isolation from the 14 day-old KMC-IMBG1 liquid hybrid culture was performed with innuSPEED bacteria/fungi DNA isolation kit (Analytik Jena AG). In parallel, total DNA samples from cellulose-based hybrid kombucha pellicle (as well as the 14 day-old pellicles produced by KMC-IMBG1 grown in sBTS, nsBTS, and BTH) were isolated from three specimens, using modified soft lyses method after blending of the pellicle (Gabor et al. 2003). The nucleic acids were quantified and qualified by a NanoDrop ND-1000 spectrophotometer (NanoDrop Technologies, Wilmington, DE).

### PCR amplification, DNA sequencing and analysis

Bacterial and yeast isolates from KMC-IMBG1 were identified by PCR amplification using standard primers 27F/1494R (AGAGTTTGATCCTGGCTCAG/TGACTGACTGAGGYTACCTTGTTACGACTT) for bacterial 16S rDNA and NL1/NL4 (GCATATCAATAAGCGGAGGAAAAG/GGTCCGTGTTTCAAGACGG) for fungal 26S rDNA amplification as it was described previously (Ogino et al. 2001; Kurtzman and Robnett 1997). More specifically, the PCR reactions for both primers were run for 35 cycles with annealing temperature 54°C for 27F/1494R and 52°C for NL1/NL4. PCR products were cleaned with UltraClean™ PCR Clean-up DNA purification kit (MoBio Laboratories). The PCR products were sequenced by the Sanger method (Sanger et al. 1977) using Big Dye Terminator Sequencing Standard Kit v3.1 (Applied Biosystems, USA) and apparatus 3130 Genetic Analyser (Applied Biosystems). The 16S rDNA sequences were binned by BLASTN search through the National Center for Biotechnology Information (NCBI) GenBank (US National Library of Medicine, Bethesda, Maryland, USA). These sequence data have been submitted to the GenBank database under an accession numbers KF908872-KF90879.

### DNA pyrosequencing

DNA sequencing has been performed by using Roche GS FLX in Inqaba Biotec (http://www.inqababiotec.co.za). Pairs of standard primers 27F 5′-AGAGTTTGATCCTGGCTCAG-3′ (Lane 1991) and 518R 5′ATTACCGCGGCTGCTGG-3′ (Muyzer et al. 1993) for 16S; and ITS1 5′-TCCGTAGGTGAACCTGCGG-3′ and ITS4 5′-TCCTCCGCTTATTGATATGC-3′ (White et al. 1990) for ITS amplification were used. Generated 16S rRNA reads were checked for chimers by using DECIPHER algorithm (Wright et al. 2012) set for analysis of short-length sequences. In total 30 putative chimeras were identified and removed from the read datasets. Quality control was performed by locally installed Fast QC program (http://www.bioinformatics.babraham.ac.uk/projects/fastqc). Poor quality reads with Phred quality score below 20 (that corresponded to p value ≥0.05) and reads shorter than 100 bp were filtered out.

### Metabarcoding dataset statistics

DNA reads obtained from the sequencer were aligned by the local BLASTN against combined NCBI 16S Microbial and GreenGenes16S databases for identification of 16S rDNA reads and against the NCBI nt-database for identification of ITS reads. The latest versions of GreenGene and NCBI databases available at the time of running of this analysis, i.e., the mid of 2014, were used in this study. The BLASTN results were merged and visualized by MEGAN 5.2.3 (Huson et al. 2011). Additionally, the BLASTN output files were searched by an in-house BioPython based script to retrieve the statistics of the top scored hits over all reads. A taxon presence in a sample was accepted, if there were at least five reads binned to this taxonomic unit. The minimum BLASTN score for taxon identification was 300. Statistics of pyrosequencing is shown in Table 1.

**Table 1:** DNA reads obtained by Roche 454 sequencing of different samples

Sample	Total number of reads before and after filtering and chimera removal	Total length, bp	Average	Min. read length^a^	Max. read length	S_obs_/S_exp_^†^
sBTS						
Pellicle: 16S	2,384/2,356	1,123,074	471	77	607	14/46
Pellicle: ITS	532/530	277,232	521	61	568	5/10
BTH						
Pellicle: 16S	2,632/2,626	1,244,214	472	47	828	14/46
Pellicle: ITS	7,888/7,783	3,303,150	418	43	561	23/87
nsBTS						
Pellicle: 16S	1,880/1,828	870,798	463	65	563	24/33
Pellicle: ITS	3,741/2,310	1,138,925	304	41	536	7/23
Hybrid KMC						
Pellicle: 16S	8,716/8,250	2,975,027	341	40	513	9/10
Pellicle: ITS	7,943/7,113	2,500,278	314	40	541	18/34
Liquid phase: 16S	6,494/6,325	2,294,949	353	40	513	16/17
Liquid phase: ITS	9,541/8,281	3,165,774	331	40	521	26/38

^a^All reads shorter than 100 bp were filtered out.

^†^ *S*_*obs*_ observed number of species including those identified by a single read, *S*_*exp*_ expected number of species according to Chao estimation (Eq. 1).

Not filtered metabarcoding data sets were deposited in the Metagenomics RAST database server (4543580.3-4543590.3).

Expected species richness of a sample was estimated according to Chao 1 equation (Chao 1984):

1 $$S_{\exp } = S_{\text{obs}} + \frac{{F_{1}^{2} }}{{2F_{2} }}$$

where S_exp_—expected species richness; S_obs_—observed number of species; F1 is the number of singletons (i.e., the number of species with only a single occurrence in the sample) and F2 is the number of doubletons (the number of species with exactly two occurrences in the sample).

Rarefication curves were estimated by counting of number of identified species after successful binning of every 200 reads. An exception was the dataset ITS_sBTS when species number increment was measured every 100 successfully binned reads because of the small size of this dataset. The binning was considered as successful if the BLASTN score was ≥300.

Distance between two metabarcode datasets was measured by the Eq. 2:

2 $$D = \sqrt{\frac{\sum\limits_{N_{\rm comb}} {\left({m_{1}} \left/{N_{1}}\right. -{m_{2}} \left/{N_{2}}\right. \right)^{2}}}{N_{\rm comb}}}$$

where N_comb_—total number of identified species in both datasets; m_1_ and m_2_—numbers of reads binned to the species *m* in the datasets 1 and 2, respectively; N_1_ and N_2_—total numbers of binned reads in the datasets 1 and 2, respectively. Distances were used to infer dendrograms of dataset diversity by using the Neighbor–joining algorithm implemented in MEGA6 (Tamura et al. 2013).

## Results

### Isolation of cultivable forms of microorganisms associated with KMC

DNA fragments amplified by PCR from DNA samples extracted from cultivable isolates of KMC-IMBG1 were binned to taxonomic units by BLASTN alignment. Members of four yeast genera *Pichia*, *Brettanomyces/Dekkera*, *Candida* and *Zygosaccharomyces*; and two bacterial genera *Gluconacetobacter* (now *Komagataeibacter* gen. nov., Yamada et al. 2012) and *Gluconobacter* were identified. On the species level there were *Komagataeibacter* sp. (99% homology to *K.xylinus* and *K.saccharivorans*), *K. intermedius, K.kombuchae*, and *Gluconobacter oxydans*. As it was revealed by culture methods, the simplest structure of KMC cultivated in sterile black tea with sugar (sBTS) composed of two yeast species of *Pichia* and *Brettanomyces/Dekkera*; and two acetobacteria: *Komagataeibacter* sp. and *K.intermedius*. In classic non-sterile sweetened black tea medium (nsBTS), KMC-IMBG1 comprised *Pichia* sp., *Dekkera anomala*, *Candida* sp., *Komagataeibacter* sp., *K. intermedius* and *Gluconobacter oxydans.* Additional yeast species *Zygosaccharomyces bailii* and acetobacterium *K.kombuchae* were isolated from the culture maintained in the sterile black tea medium with honey (BTH). In the hybrid kombucha culture grown in BTH mixed with cabbage brine, several atypical bacterial species have been identified including *Bacillus subtilis*, *B. pumilis (Firmicutes)* and *Microbacterium* sp. (*Actinobacteria*).

At the same time, the confocal scanning laser microscopy revealed a higher level of diversity of KMC-IMBG1 especially those associated with the cellulose 3D web (Additional file 1: Figure S1). It was hypothesized that uncultivable microbial organisms might be abundant in this network. Particularly, there were peculiar long cells observed during the dormancy state (Additional file 1: Figure S1b), which were dissimilar to any cultivable bacteria (Puspita et al. 2012). To overcome the problem of identification of uncultivable representatives of KMC-IMBG1, a metabarcoding approach has been used.

### Metabarcoding analysis of KMC-IMBG1 grown in different conditions

**Figure 1:**
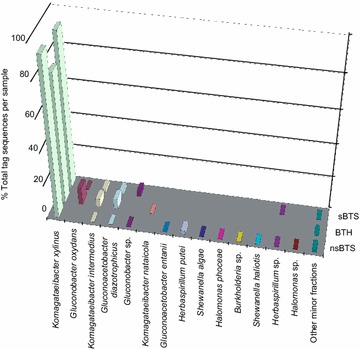
Profiles of bacterial species of KMC-IMBG1 grown in sterile black tea with sugar (sBTS), non-sterile black tea with honey (BTH) and non-sterile black tea with sugar (nsBTS) identified by binning of 16S rDNA reads.

According to the results of analysis of metabarcodes, the acetobacteria of *Komagataeibacter* and *Gluconobacter* genera (α-*Proteobacteria*) dominated in KMC-IMBG1 grown in sBTS, nsBTS and BTH with a few other bacterial species of *Komagataeibacter*. *K.xylinus* prevailed in all analysed bacteriomes (77.7–96.8%); *K.intermedius* reached up to 4.9%, and *Gluconobacter* spp., most of which belonged to *G.oxydans*, composed up to 10% of bacterial community in BTH. However, when grown in nsBTS, the proportion of *Gluconobacter* decreased 50-folds (Figure 1). This can be explained by the preferred consumption of different sugars present in honey (Mandal and Mandal 2011). Estimated richness of bacterial and fungal species of KMC is shown in Table 1. Interestingly, *Gluconoacetobacter diazotrophicus* known as an obligate sugarcane endophyte (Baldani et al. 1997) was constantly present in all variants of KMC studied in this work; however, this species was not isolated by culture-dependent method. *Herbaspirillum* spp. and *Halomonas* spp. were a minor, but permanent component in KMC-IMBG1 grown in all the different conditions. Presence of *Halomonas* sp. was also reported in kombucha microbiota revealed in the metabarcoding study by Shade (2011). The minor fractions of the KMC-IMBG1 were represented by several occasional *Firmicutes*, β-and γ-*Proteobacteria* (see Figure 1).

**Figure 2:**
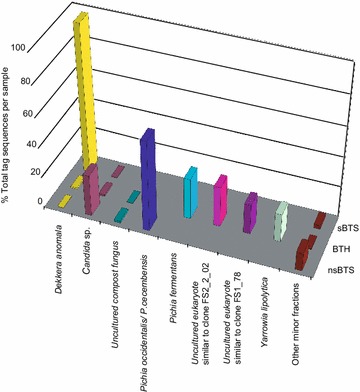
Profiles of yeast species in KMC-IMBG1 grown in sterile black tea with sugar (sBTS), sterile black tea with honey (BTH) and non-sterile black tea with sugar (nsBTS) identified by binning of ITS reads.

The metabarcoding showed that KMC-IMBG1 comprised yeast species belonging to *Pichia*, *Brettanomyces/Dekkera*, *Candida* and *Saccharomyces* genera, as well as unknown OTUs similar to ‘compost fungus’ and ‘unknown yeast’. The yeast composition widely varied in different cultures. The major yeast species of KMC-IMBG1 grown in sBTS was *Dekkera anomala*. *Pichia fermentas* was abundant in BTH, and *Pichia occidentalis* (former *Issatchenkia occidentalis*) was the most frequent in nsBTS (Figure 2). This observation suggested that the domination of that or another yeast species significantly depended on the cultivation conditions at much higher extend than it was observed for the core bacterial community (see Figures 1, 2). It was remarkable that an uncultured unknown fungal species identified as ‘compost fungus’ was the most abundant in BTH and to some extend in nsBTS.

### Metabarcoding analysis of the hybrid kombucha culture

**Figure 3:**
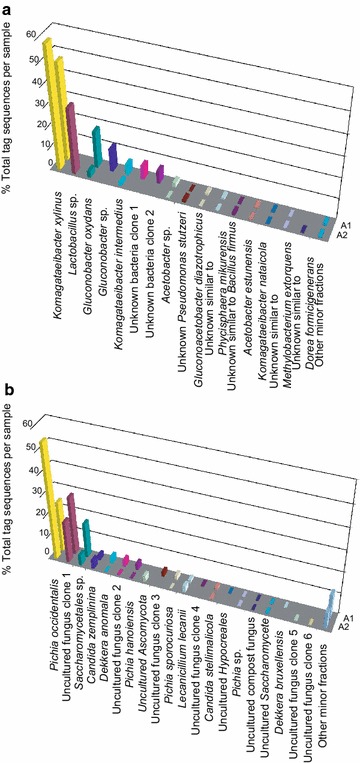
Normalized abundance of the most frequent OTUs of KMC identified by BLASTN in **a** 16S rDNA and **b** ITS reads. A1—liquid phase of the culture; A2—cellulose based biofilm. Numbers of identified reads were normalized by the total numbers of reads in the samples.

Bacterial and yeast communities of the hybrid KMC-IMBG1 grown in a mixture of filter-sterilized BTH with added sweetened fermented cabbage brine were expectedly much more diverse (Figure 3). *K.xylinus* was a dominant bacterial species. Lactobacilli, which probably originated from the cabbage brine and remained here in series of passages, were abundant in the hybrid KMC-IMBG1 pellicles. *Lactobacillus* spp. isolates were reported before as indispensable kombucha community members (Marsh et al. 2014). Lactobacilli are known also as the indigenous inhabitants of fermented cabbage (Jung et al. 2011). *L.plantarum* causes fermentation of cabbage carbohydrates to the lactic acid or acetic acid.

Several bacterial OTUs failed with taxonomic affiliation because of lack of appropriate reference sequences in the searched databases (Figure 3a). Yeast DNA barcoding discovered a much higher number of OTUs in pellicles and cultural liquid in the hybrid kombucha culture as compared to the parental KMC-IMBG1. *P.occidentalis*/*P.cecembensis* were the dominant yeast species the same as in nsBTS (Figure 3b). Many OTUs were not affiliated to any taxonomic units because of a weak sequence similarity, or they showed similarity to unknown microorganisms. Anyway, even the weak similarity was consistently against the same reference sequences that suggested that the total number of species in KMC-IMBG1 was limited but many of them still remained unknown.

### Comparative analysis of microbiomes produced by KMC under different microenvironments

**Figure 4:**
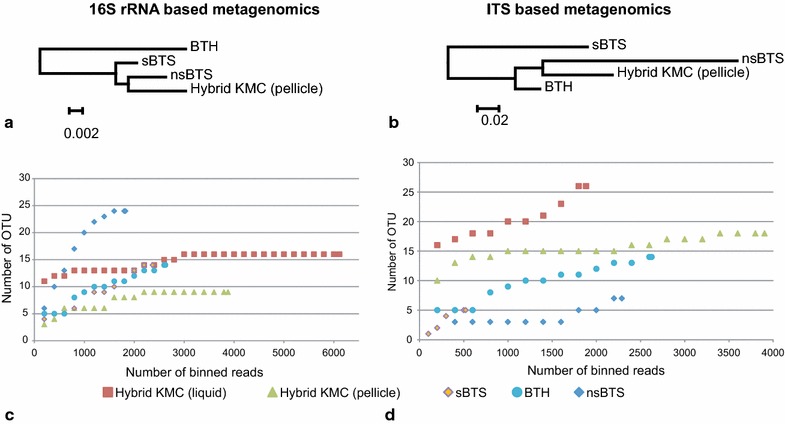
Statistical analysis of metabarcode datasets based on 16S rDNA (parts **a** and **c**) and ITS (pats **b** and **d**) amplicons. Dendrograms in **a** and **b** of diversity of datasets were built by neighbor–joining algorithm based on distance tables calculated by Eq. 2. Parts **c** and **d** represent rarefication curves.

Rarefication curves for studied metabarcode datasets and dendrograms representing species diversity of KMC pellicle grown at different conditions are shown in Figure 4. Remarkably, fungal biomes of KMC varied to a much higher extend depending on the growth conditions than the bacterial component (see the scaling bars in dendrograms in Figure 4). The biggest number of bacterial OUTs, which were singletons or represented only by a few reads, was observed in KMC grown in non-sterile conditions (nsBTS). It is reflected in the steepness of the corresponding rarefication curve in Figure 4. Interestingly, the richness of fungal species of the sample was depleted, that may be explained by presence of *Bacillus* and *Pseudomonas*, which might synthesize antifungal antibiotics. Nevertheless, the bacterial core component in nsBTS remained the same. The biggest alteration in the KMC bacteriome structure was observed in BTH (Figure 4a) caused probably by honey addition. Microbial composition of the hybrid KMC including both the bacterial and fungal components showed a higher level of stability as the rarefication curves calculated for this community has got faster the saturation level (Figure 4c, d).

## Discussion

This study showed that KMC-IMBG1 is quite flexible and variable community. The main highlight of this study was that KMC-IMBG1 grown on different sterile and non-sterile media produced a stable core microbiome comprising acetobacteria and few associated strains of yeast species, and a number of accessory species, which may or may not occur at different conditions. The core part of KMC-IMBG1 probably is critical for functioning of the whole community and might be responsible for recovery of the community after disturbance. In addition to prevalent community members, several minor but permanently occurring bacterial species of KMC-IMBG1 were also discovered. Among them there were *Herbaspirillum* spp. and *Halomonas* spp. These organisms were identified by binning the DNA reads originated from KMC grown in sterile and non-sterile media. In other studies on species composition of kombucha from North American and Ireland ecotypes these species were not reported (Marsh et al. 2014). Another disagreement with the report by Marsh et al. (2014) was that according to these authors the highest diversity of micro-flora was associated with the cellulose pellicle. In Figure 4c, d it is seen in rarefication curves of the hybrid KMC-IMBG1 that both fungal and bacterial micro-flora of pellicle was more stable and less rich in different species than that from the liquid phase. Further research is needed to uncover the role of the core and accessory members of KMC, including the uncultivable bacteria, and how they contribute to stabilizing the community and gaining its biologically active. The ability to modify KMC is of practical importance as a possible approach to improve the medicinal and biotechnological properties of the kombucha products (Kozyrovska et al. 2012). This work is the promising first step to design efficient and safe probiotics and synbiotics based on synthetic KMC communities of beneficial and harmless microbial species. It was hypothesized that the positive activity of kombucha probiotic on human health may be improved and extended by ‘domestication’ in the kombucha of other probiotic bacteria, e.g., lactobacilli, which in the current study most likely were recruited by KMC from the cabbage brine. There is still much to be discovered about bacteria-yeast communal interrelationships and their impact on the human microbiota. It also may be concluded that the DNA metabarcoding based on NGS is the best choice for profiling of complex microbial communities of fermented products.

## Additional files

Additional file 1:
**Figure S1.** Confocal scanning laser microscopy images of a cross section of cellulose-based pellicle produced by KMC in a sugared black tea, showing a variety of both bacteria and yeast cell morphotypes (**a**); cells of unusual morphology (a long shape), which may indicate the existence of dormant uncultivable microbial (sub)populations (**b**). Cellulose and yeast cells stained with calcofluor (*a* *blue signal*), bacterial cells and proteins stained with thiazine red (*a yellow signal*). *Scale bar* is 10 μm.

## Abbreviations

KMC: kombucha microbial community
KMC-IMBG1: kombucha microbial community variant from Ukrainian collection
NGS: next generation sequencing
ITS: internal transcribed spacer
sBTS: kombucha culture grown on sterile black tea with sucrose
nsBTS: kombucha culture grown on non-sterile black tea with sucrose
BTH: kombucha culture grown on sterile black tea with honey
