# Supplementary material for: Metabarcoding of the kombucha microbial community grown in different microenvironments
Source: AMB Express. 2015 Jun 11;5:35. doi: 10.1186/s13568-015-0124-5 (PMC4467805; doi:10.1186/s13568-015-0124-5)
Supplement: Additional file 1: — Figure S1. Confocal scanning laser microscopy images of a cross section of cellulose-based pellicle produced by KMC in a sugared black tea, showing a variety of both bacteria and yeast cell morphotypes (a); cells of unusual morphology (a long shape), which may indicate the existence of dormant uncultivable microbial (sub)populations (b). Cellulose and yeast cells stained with calcofluor (a blue signal), bacterial cells and proteins stained with thiazine red (a yellow signal). Scale bar is 10 μm. [file 13568_2015_124_MOESM1_ESM.pdf]

Applied Microbiology and Biotechnology;

Metabarcoding of the kombucha microbial community grown in different microenvironments;

Oleg N Reva[*1], Iryna E Zaets[2], Leonid P Ovcharenko[2], Olga E Kukharenko[2], Switlana P Shpylova[2], Olga V Podolich[2], Jean-Pierre de Vera[3], Natalia O Kozyrovska[2].

[1]Bioinformatics and Computational Biology Unit, Department of Biochemistry, University of Pretoria, Lynnwood road, Hillcrest, Pretoria, 0002, South Africa;
[2]Institute of Molecular Biology & Genetics of National Academy of Sciences of Ukraine, Acad. Zabolotnoho str., 150, 03680 Kyiv, Ukraine;
[3]Institute of Planetary Science, DLR, Rutherfordstr. 2, D-12489 Berlin, Germany;

[*]Corresponding author, oleg.reva@up.ac.za; tel. +27-12420-5810; fax +27-12420-5800;

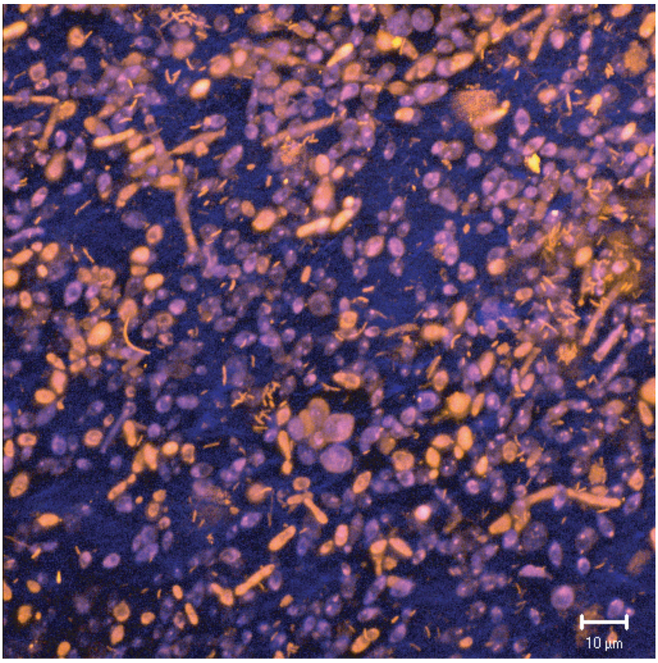

A

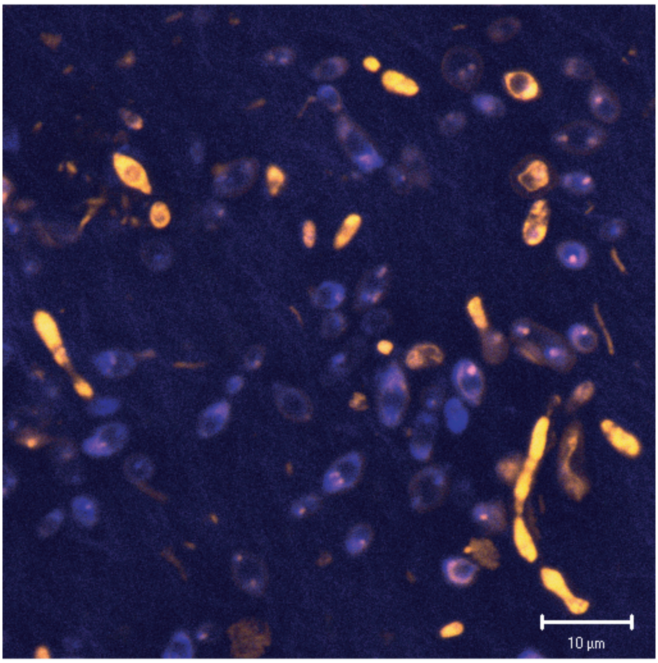

B

Fig. S1. Confocal scanning laser microscopy images of a cross section of cellulose-based pellicle produced by KMC in a sugared black tea, showing a variety of both bacteria and yeast cell morphotypes (A); cells of unusual morphology (a long shape), which may indicate the existence of dormant uncultivable microbial (sub)populations (B). Cellulose and yeast cells stained with calcofluor (a blue signal), bacterial cells and proteins stained with thiazine red (a yellow signal). Scale bar is 10 µm.
